# Critical emergency medicine unit: a new model to mitigate critically ill patient boarding in emergency department

**DOI:** 10.1186/s44158-025-00262-x

**Published:** 2025-07-10

**Authors:** Felice Urso, Daniele Catalano, Ileana Suprina Petrovic, Enrico Boero, Paola Berchialla, Luigi Vetrugno, Daniela Silengo

**Affiliations:** 1 Anesthesia and Intensive Care Unit, Ospedale San Giovanni Bosco, Turin, Italy; 2 Department of Clinical and Biological Sciences, University of Turin, Turin, Italy; 3 Department of Anesthesiology, Critical Care Medicine and Emergency, SS. Annunziata Hospital, Chieti, Italy

**Keywords:** Critical care, Emergency medicine, Anesthesiology, Hospital mortality, Lengths of stay, Emergency service, Hospital/organization and administration, ICU

## Abstract

**Background:**

Boarding of critically ill patients in the emergency department (ED) is an emerging problem that increases mortality. We have developed a “CREM Unit (critical emergency medicine unit)” led by an anesthetist-intensivist who manages critical patients directly in the ED. This study aims to assess whether the CREM Unit is an effective model for mitigating the boarding of critical patients in the ED and the impact of this on mortality.

**Method:**

This is a retrospective observational study. We collected all patients assigned to the CREM Unit from January 1, 2019, to December 31, 2021. As our primary endpoints, we calculated ED boarding rate and the impact of boarding time on mortality. As a secondary endpoint, we compared observed 28-day mortality to Simplified Acute Physiology Score (SAPS II) predicted mortality.

**Results:**

Patients managed by the CREM unit were 127 in 2019, 181 in 2020, and 206 in 2021, with a clear upward trend, for a total of 514 patients (*p* < 0.001). Overall boarding rate was 13.9%, and length of stay in ED was not associated with an increased mortality (*p* = 0.399). Observed mortality was compared with expected mortality, estimated from the SAPS II score for a group of inpatients (*n* = 295). Moreover, the median value of SAPS II for inpatients was 54 (40.5–69.0), with an expected mortality of 55.3%, while the observed mortality was 36.8% (95% *CI* 31.9% to 42.1%, *p* < 0.0001).

**Conclusions:**

Over the years, the number of patients assigned to the CREM Unit has grown steadily. These data suggest that the CREM Unit cares for a significant number of critically ill patients and could have a well-defined role both in keeping their boarding low and may contribute to reducing its impact on mortality.

**Supplementary Information:**

The online version contains supplementary material available at 10.1186/s44158-025-00262-x.

## Highlights

∙ CREM Unit boarding time is no longer an independent risk factor for a critically ill patient’s mortality.

∙ CREM Unit cares for a significant number of critically ill patients and has a well-defined role both in keeping their boarding low and reducing its impact on mortality.

∙ Strength of the ICU-based model lies in the longitudinal approach, capable of guaranteeing an early resuscitative approach, individualization of invasive monitoring, and early medical-surgical therapeutic strategies.

**Supplementary Information:**

The online version contains supplementary material available at 10.1186/s44158-025-00262-x.

## Introduction

**Fig. 1:**
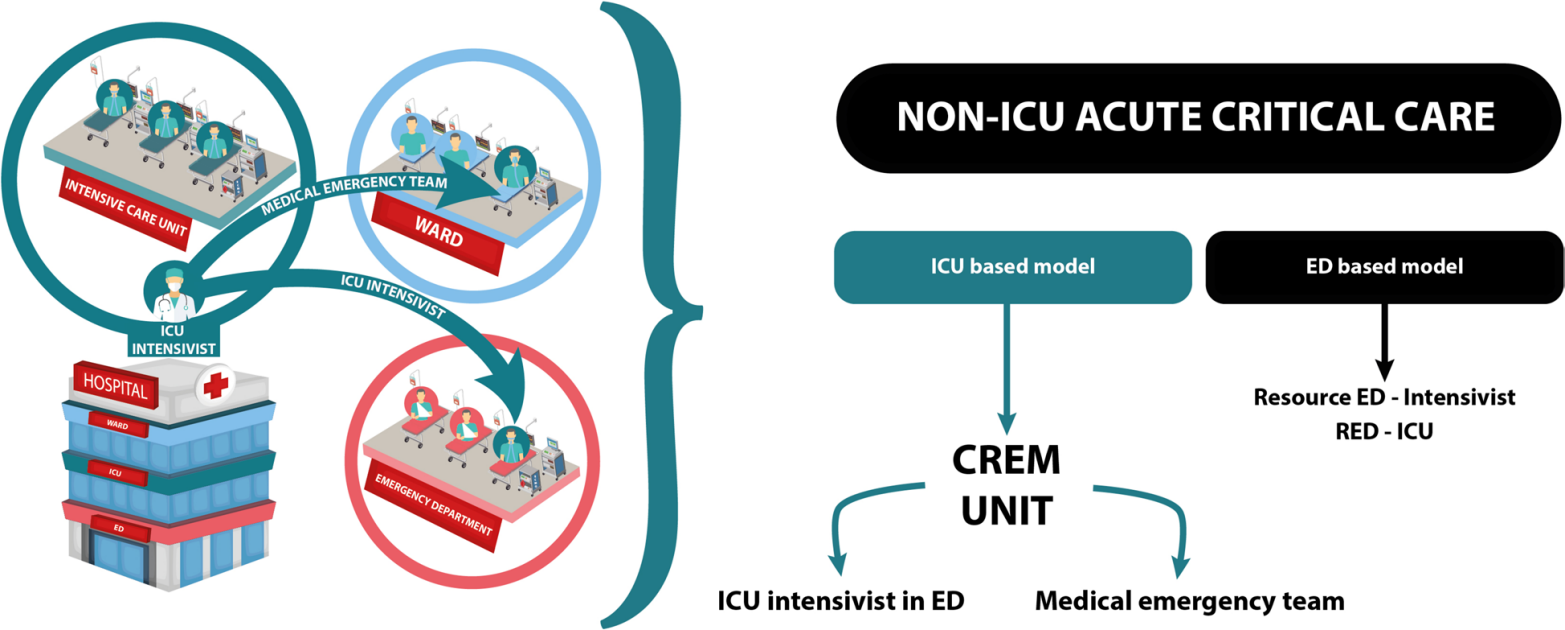
Model of intensive care unit (ICU) intensivist caring for critically ill patients outside the ICU

In recent years, overcrowding and boarding in the emergency department (ED) have increased considerably. Combined with the scarcity of intensive care unit (ICU) beds, these phenomena have led to the development of strategies to manage acute critically ill patients in the ED and mitigate the adverse effects of prolonged boarding time on patients’ outcomes [1, 2]. No universally accepted definition of ED boarding has yet been adopted. Nevertheless, adverse outcomes are more frequent in critically ill patients with an ED stay longer than 6 h [3]. Even though precise data about boarding are limited by the lack of standardization of the definition, it is estimated to range from 2.1% to 87.6% [4]. Many reports in the literature highlight how boarding in EDs under resource-limited conditions increases both morbidity and mortality [4–6]. For this reason, many institutions have developed strategies to mitigate its impact. In the USA, this is mainly done by using an ED-based model relying on the presence of either a Critical Care Resource Intensivist or a Red-ICU (Fig. 1).

In Europe, however, a model called “ICU without walls” has recently been developed. In this model, critically ill patients in the ED receive medical care directly from the intensive care medicine (ICM) specialist. Alongside the well-known perioperative management, ICM, and pain medicine, the new critical emergency medicine (CREM) has been included as a required competence of European specialists by the European Board of Anaesthesiology [7]. In particular, CREM is the discipline that deals with the immediate need for life support and resuscitation of critically ill patients and is distinguished from the much broader spectrum of emergency medicine (EM), which deals generically with all acute patients [8]. This type of training allows for a “longitudinal” approach that prioritizes the severity of illness in the critically ill patient, going beyond the resuscitation phase and considering the patient’s trajectory. Early assessment of resuscitation by the anesthesiologist is helpful in setting the subsequent treatment of critically ill patients [9].

Over the past 20 years, our ED has always been able to count on consultation from the Anesthesia and Intensive Care Unit Department 24/7, and this has allowed the creation of an excellent relationship and collaboration with emergency physicians and the other ED staff. As well as other institutions in recent years, we have considered Safar’s Critical Care Unit as a concept rather than a location [1, 10] and have established an anesthesiologist-led team operating in the emergency department and, if necessary, on any inhospital emergency as part of the medical emergency team (MET). Based on this, we have tried to define a new organizational model in our hospital inspired by the “ICU without walls” concept [11]. We refer to this model as the critical emergency medicine unit (CREM Unit). With the introduction of the CREM Unit, the anesthesiologist manages patients as an “ICU intensivist” in a setting considered to be a hybrid ICU model [1], using beds within the ED that can be transformed into high-intensity care beds at any time with all the necessary equipment already available on site.

There are few studies addressing the impact of an anesthetist-led team on the well-known boarding phenomenon. The management of the critical patient in the ED also varies depending on hospital protocols, patient influx, and the availability of emergency medical personnel. This certainly represents a gap in our knowledge of the effectiveness of certain models.

Finally, there are no studies in the literature that address the phenomenon of boarding in the Italian reality and the related mitigation strategies. We believe that the added value of the longitudinal model lies precisely in the critical care that the intensivist can provide during the acute management of the critical patient directly in the emergency department, thus addressing the impact that this may have on boarding in terms of morbidity and mortality.

This study aims to evaluate how this organizational model has been incorporated into our ED and its effectiveness in mitigating the critically ill patient boarding effect.

## Methods

### Study design and period

We performed a retrospective cohort analysis of electronic health records (EHR) of patients handled by the CREM Unit from January 1, 2019, to December 31, 2021, in San Giovanni Bosco Hospital, Turin, Italy.

The triage nurses assign a five-level priority code based on clinical severity. The red code (level 1) implies the clinical scenario of an emergency. In contrast, yellow, higher-green, lower-green, and white codes (levels 2 to 5) indicate progressively lower priority of access to medical evaluation. For the sake of the CREM Unit, patients were assigned to it directly by the triage nurse based on three criteria:

- Any out-of-hospital cardiac arrest (OHCA)
- Invasive mechanically ventilated (IMV) patients from emergency medical services (EMS)
- Critical traumatic injuries

Patients can also be assigned to the CREM Unit by any physician in case of subsequent clinical deterioration requiring resuscitative evaluation. A third group of patients was transferred from spoke-type hospitals to perform emergent procedures or surgery (i.e., neurosurgery, vascular surgery, interventional radiology procedures).

### Primary and secondary outcomes

**Fig. 2:**
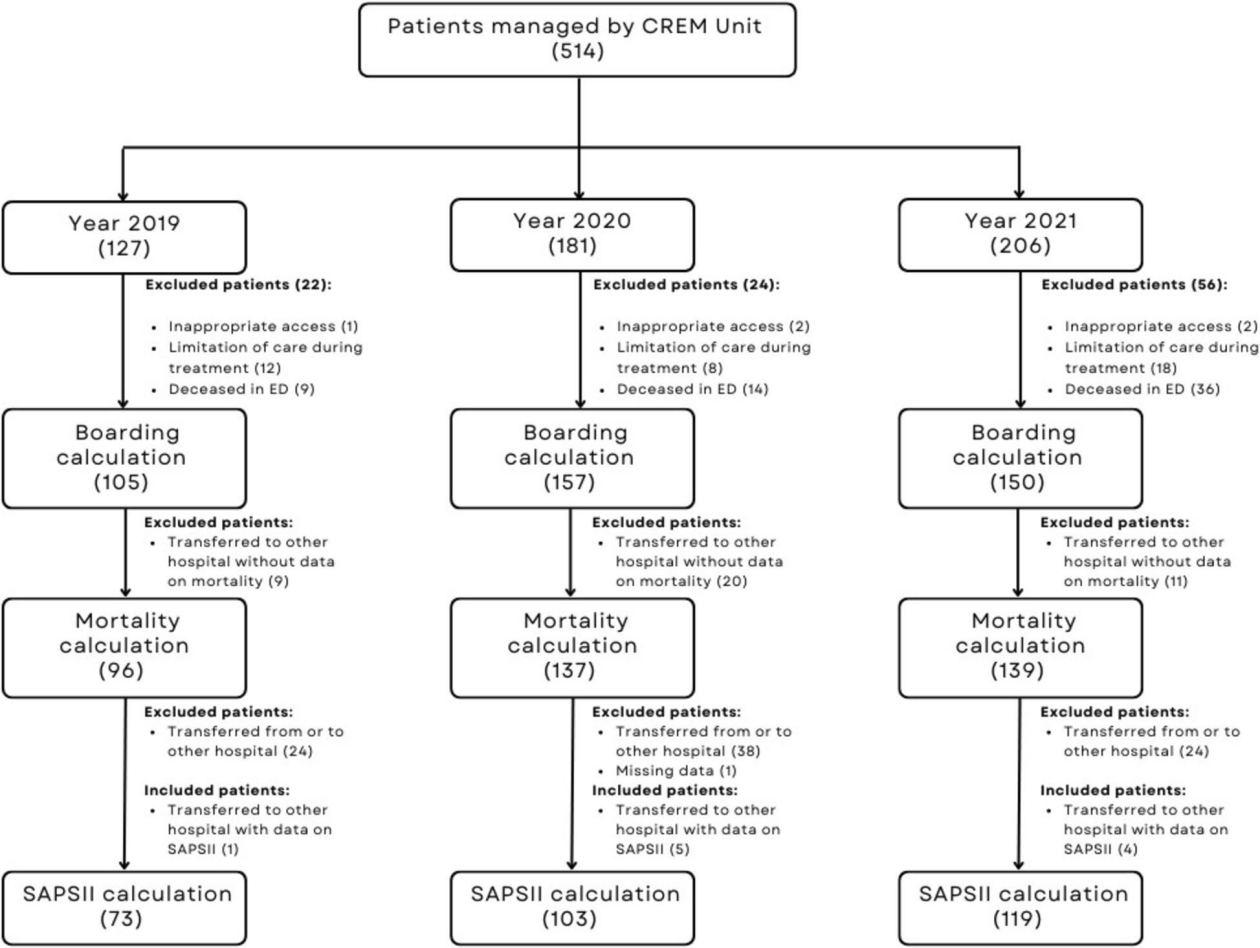
Flow diagram of the retrospective cohort showing the absolute frequency of patients managed by CREM Unit per year and the proportion of patients for whom boarding, mortality, and SAPS II were calculated

As our primary endpoint, we calculated ED boarding rate and the impact of boarding time on mortality. For patients assigned to the CREM Unit directly from triage, ED boarding time was calculated from the time of triage assessment to the time of transfer to the intensive care unit; for patients assigned to the CREM Unit due to clinical deterioration, ED boarding time was calculated from the time of the first request for intensivist assessment. As our secondary endpoint, we compared observed 28-day mortality to Simplified Acute Physiology Score (SAPS II) predicted mortality. Patients for whom reliable data regarding SAPS II and 28-day outcome were not available were excluded from the comparison between expected and observed mortality. In fact, patients transferred from other hospitals did not have precise SAPS II data, and patients who died within the first 24 h would not have had a meaningful SAPS II score. Furthermore, for some patients who were managed, it was not possible to obtain a definitive 28-day outcome as they had been transferred to hospitals outside the hospital network. Patient flow diagram is shown in Fig. 2.

### Patients’ characteristics and variables

We described the number of patients treated, their characteristics, the causes of hospitalization, and the length of their stay in the ED over the first 3 years of the CREM unit’s activity.

The time of admission to the ED and time of transfer to each specific ward (ICU, OR, high-dependency units, stroke unit) were registered.

Boarding of patients managed by the CREM Unit for worsening clinical status following evaluation by other physicians was assessed from the time of resuscitative assessment performed by the ICU intensivist. Investigators registered the medical admission causes based on the diagnosis at patient discharge contained in EHR. Transferred patients were considered for boarding calculation.

Patients who were treated upon arrival but eventually died in the ED were considered in the overall mortality rate. On the opposite, patients who arrived in extremely critical condition and died shortly after and patients who were put on palliative care were excluded. Mortality data were obtained by analyzing the EHR records available for the overall sample. We calculated the expected mortality based on the SAPS II score and compared it to the observed mortality rate. Observed mortality was then calculated for the patients admitted to our hospital, excluding those who did not survive in the emergency department and whose 28-day outcome data were missing.

Since studies have demonstrated the prognostic value of SAPS II in predicting inhospital mortality [12], the score was calculated retrospectively over 24 h for all patients managed by the CREM Unit and subsequently admitted to our hospital, based on available clinical documentation reviewed by the authors. We excluded from the SAPS II calculation all patients who died in the emergency room, patients transferred to other hospitals after initial assessment except for cases with full data, patients transferred from spoke hospitals, and patients whose clinical records were unavailable. We also report the total Sequential Organ Failure Assessment (SOFA) score for comparison, calculated as the sum of the highest value for each subscale (cardiovascular, respiratory, hematologic, neurologic, hepatic, and renal) in the first 24 h of care. The predictive models referring to ED length of stay and mortality were formulated based on the available data. The missing data are related to the prognosis of patients who were transferred to hospitals outside the local health network. No missing data were imputed or retrospectively estimated. The only missing values in the dataset related to patients’ prognoses. Predictive models regarding ED length of stay and mortality were analyzed based on SAPS II and several other relevant variables (type of admission to the CREM Unit, age, gender, and severity assessed at triage).

### Data handling and statistical analysis

The City of Turin Institutional Review Board approved the study with protocol number 0059874. This study was conducted in accordance with the Declaration of Helsinki.

The EHR software used in the ED enabled fully digitalized evaluation of patient records and subsequent data handling and analysis (JHiS, S.In.Co.S. Applications S.R.L., Turin).

Continuous variables were reported as means and standard deviation (SD) or median with interquartile range (IQR) as appropriate and categorical variables as numbers and percentages.

Evaluation of predictors of boarding and mortality was first assessed at the univariate level. Restricted cubic splines were modeled to assess the nonlinear effect, and significance was tested by the Wald chi-square. Significance was set at 0.05. Age and SAPS II showed a posterior significant probability of influencing our outcomes and were retained in the model. We computed 95% confidence intervals for observed mortality using the Wilson approximation and for predicted probabilities using the bootstrap percentile method. All analyses were carried out using R 4.0.0 [R Core Team (2022), R: A Language and Environment for Statistical Computing, R Foundation for Statistical Computing, Vienna, Austria].

## Results

**Fig. 3:**
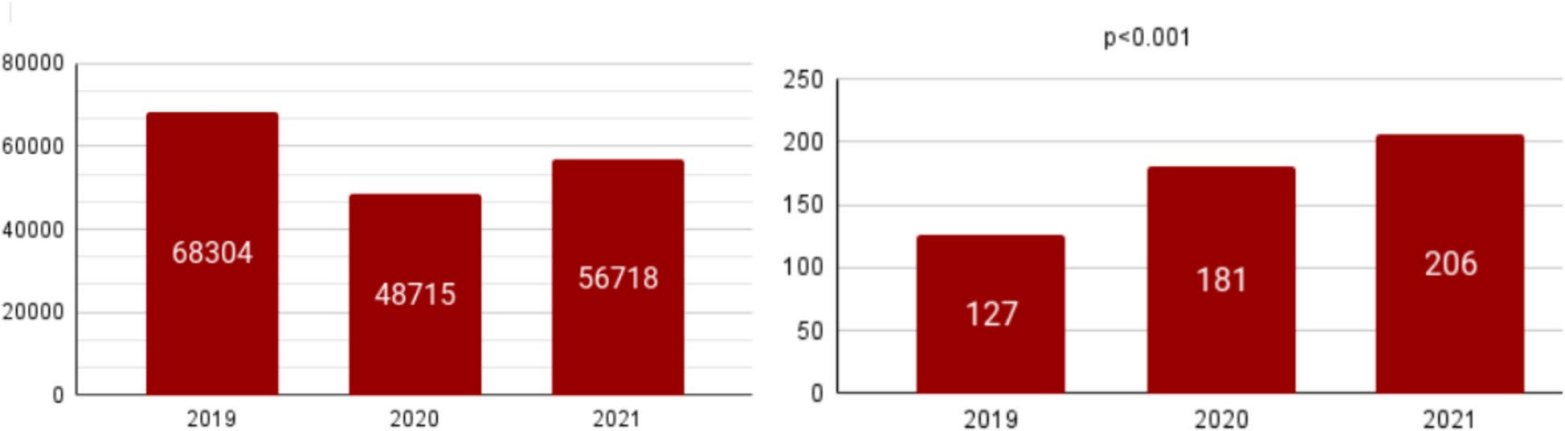
Absolute frequencies of emergency department (ED)-admitted patients (**A**) and absolute frequencies of CREM Unit-managed patients (**B**)

The total number of patients managed by the ED of San Giovanni Bosco between Jan 1, 2019, and Dec 31, 2021, was 173,737. A total of 514 cases of patients assigned to the CREM Unit were evaluated during the study period: 127 patients were managed by the CREM Unit in 2019, 181 in 2020, and 206 in 2021, with a significant upward trend (*p* < 0.001) (Fig. 3). Of these 514 patients, 230 patients were directly assigned by a triage nurse to the CREM Unit upon arrival, showing a constant increase over the year (*p* < 0.001), whereas 286 were acquired following clinical deterioration (Table 1).

**Table 1:** Summary of characteristics of our cohort of patients including demographic data, triage admission type, clinical emergency category, and severity scores

Patients managed by CREM Unit	Overall	Years			*p*
		2019	2020	2021	
Overall *N* (%)	514 (100)	127 (24.7)	181 (35.2)	206 (40.1)	
Criteria for CREM Unit admission *N* (%)					< 0.001
Directly assigned to CREM Unit	234 (45.5)	52 (40.9)	64 (35.4)	118 (57.3)	
Transferred from other hospitals	83 (16.1)	24 (18.9)	34 (18.8)	25 (12.1)	
Worsening patients	197 (38.3)	51 (40.2)	83 (45.9)	63 (30.6)	
Demographic					
Mean age mean (SD)	61.25 (16.60)	63.28 (15.92)	61.87 (15.15)	59.46 (18.06)	0.10
Male *N* (%)	333 (64.8)	82 (64.6)	115 (63.5)	136 (66.0)	0.87
Ethnicity *N* (%)					0.38
Caucasian	481 (93.6)	123 (96.9)	166 (91.7)	192 (93.2)	
African	30 (5.8)	4 (3.1)	14 (7.7)	12 (5.8)	
Asian	3 (0.6)	0 (0.0)	1 (0.6)	2 (1.0)	
Triage priority *N* (%)					0.006
Red code	402 (78.2)	101 (79.5)	125 (69.1)	176 (85.4)	
Yellow code	74 (14.4)	18 (14.2)	34 (18.8)	22 (10.7)	
Lower green	9 (1.8)	3 (2.4)	4 (2.2)	2 (1.0)	
Higher-Green	29 (5.6)	5 (3.9)	18 (9.9)	6 (2.9)	
Admission diagnosis *N* (%)					0.018
Neurologic emergency	166 (32.3)	40 (31.5)	54 (29.8)	72 (35.0)	
Cardiovascular emergency	102 (19.8)	22 (17.3)	37 (20.4)	43 (20.9)	
Pulmonary emergency	96 (18.7)	18 (14.2)	51 (28.2)	27 (13.1)	
Traumatic injury	84 (16.3)	25 (19.7)	20 (11.0)	39 (18.9)	
Intoxication	17 (3.3)	4 (3.1)	4 (2.2)	9 (4.4)	
Septic shock	11 (2.1)	6 (4.7)	3 (1.7)	2 (1.0)	
Endocrine and metabolic emergency	11 (2.1)	3 (2.4)	5 (2.8)	3 (1.5)	
Other	22 (4.3)	8 (6.3)	6 (3.3)	8 (3.9)	
Undifferentiated	5 (1.0)	1 (0.8)	1 (0.6)	3 (1.5)	
Severity score (*N* = 295)					
SAPS II median [IQR]	54 [40.5–69.0]	59 [41–80]	50 [39–68]	54 [41–66]	0.147
SOFA median [IQR]	8.00 [5.00–11.00]	9 [5–12]	6 [4–10]	8 [5.5–10.5]	0.076

The average age of our population was 61 years (*SD* 16.6), and 333 (64.8%) of them were male, with no difference during the study period (*p* = 0.87). The admission triage code of patients assigned to the CREM Unit was red in 78.2% of cases, yellow in 14.4%, higher green in 1.8%, and lower green in 5.6%. The most frequent causes of access to the CREM Unit were neurological emergencies (32.3%), cardiovascular emergencies (19.8%), respiratory failure (18.7%), and traumatic injuries (16.3%). Less frequent causes were intoxication (3.3%), septic shock (2.1%), and endocrine/metabolic emergencies (2.1%). Other less common causes (4.3%) were obstetric emergencies and situations requiring emergent procedures, such as gastrointestinal bleeding and urgent airway procedures. Sometimes, the diagnosis was not known or undifferentiated (1%).

The absolute incidence of boarding in the whole cohort of patients managed by the CREM Unit was 13.9%. The median length of stay in the ED was 144 min (*IQR* 75–245) (Table 2).

**Table 2:** Summary of destination ward, mortality of admitted patients, and the incidence of boarding (data presented as absolute and relative frequency unless otherwise noted)

Destination of managed patients by CREM Unit	Overall		Years	
		2019	2020	2021
Overall *N* (%)	514 (100)	127 (24.7)	181 (35.2)	206 (40.1)
Admitted to intensive care unit	183 (35.6)	50 (39.4)	63 (34.8)	70 (34)
Moved to operating room for emergency procedure	165 (32.1)	42 (33.1)	62 (34.3)	61 (30)
Deceased in ED	102 (19.8)	22 (17.3)	24 (13.3)	56 (27.2)
Transferred to other hospitals	45 (8.8)	11 (8.7)	24 (13.3)	10 (4.9)
Admitted to high-dependency unit	16 (3.1)	2 (1.6)	7 (3.9)	7 (3.4)
Admitted to stroke unit	1 (0.2)	0	0	1 (0.5)
Home discharged	2 (0.4)	0	1 (0.6)	1 (0.5)
Overall 28-day mortality	Overall		Years	
		2019	2020	2021
Overall *N* (%)	372 (100)	96 (25.8)	137 (36.8)	139 (37.4)
All patients *N* (%)	137 (36.8)	33 (34.4)	49 (35.8)	55 (39.6)
Expected mortality on SAPS II formula (%)	55.3	66.1	46.1	55.3
Incidence of boarding of hospitalized patients by CREM Unit	Overall		Years	
		2019	2020	2021
Overall *N* (%)	411 (100)	105 (25.6)	156 (38.0)	150 (36.5)
All patients	57 (13.9)	19 (18.1)	18 (11.5)	20 (13.3)
ICU	37 (20.2)	12 (24)	10 (15.9)	15 (21.4)
Operating room	4 (2.4)	2 (4.8)	0	2 (3.3)
High-dependency unit	5 (31.3)	1 (50)	2 (28.6)	2 (28.6)
Stroke unit	1 (100)	0	0	1 (100)
Transferred patient	10 (22.2)	4 (36.4)	6 (25)	0
Median time spent in ED (min) median [IQR]	144 [77–248]	136 [69–246]	134 [72–232]	157 [93–259]

The incidence of boarding stratified by year was 18.1% in 2019, 11.5% in 2020, and 13.4% in 2021. A total of 183 patients were admitted to the ICU after being managed by the CREM Unit; of them, 37 (20.2%) had an ED stay of more than 6 h from arrival or CREM Unit activation to ICU admission. On the other hand, 165 patients required emergency surgery or angiographic procedures, and 4 (2.4%) patients in this group experienced boarding. A total of 16 patients were admitted to HDU, 1 patient was admitted to the stroke unit, and 45 patients (8.7%) were transferred to other hospitals. The group of patients transferred to HDU had a boarding rate of 31.3%.

**Fig. 4:**
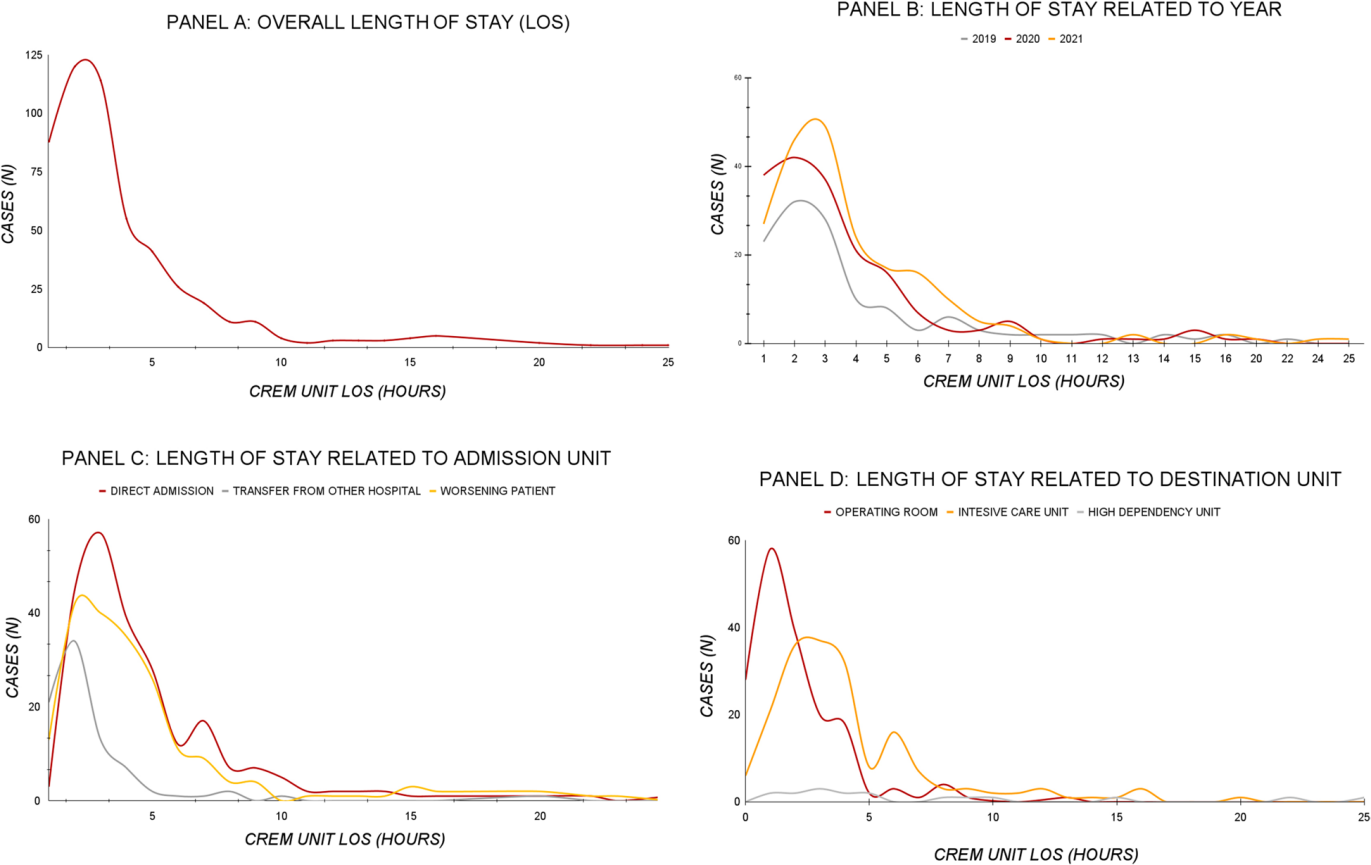
CREM Unit length of stay (LOS) shown as hours with their frequencies. **A** Overall patient data. **B** LOS stratified by year. **C** LOS stratified by unit of admission. **D** LOS stratified by unit of destination

Characteristics and outcomes of the study group stratified by CREM Unit admission are shown in Table 3. The group of patients on direct admission had a boarding rate of 19.6%, and that of patients admitted for clinical worsening had an incidence of boarding of 13.7%. Patients transferred from other hospitals had a boarding rate of 3.8% (*p* = 0.004) (Fig. 4).

**Table 3:** Summary of characteristics of included cases and outcome by CREM Unit admission (data presented as absolute and relative frequency unless otherwise noted)

Patients managed by CREM Unit	Overall	CREM Unit admission			*p*
		Direct admission	Transferred from other hospital	Worsening patient	
Overall *N* (%)	514 (100)	234 (45.5)	83 (16.1)	197 (38.3)	
Demographic					
Mean age mean (SD)	61.25 (16.6)	60.27 (17.94)	64.92 (14.51)	60.87 (15.61)	0.084
Male *N* (%)	333 (64.8)	148 (63.2)	50 (60.2)	135 (68.5)	0.332
Severity score (*N* = 295)					
SAPS II median [IQR]	54 [40.5–69.0]	59 [46.25–71.75]	-	49 [36–66]	0.002
SOFA median [IQR]	8.00 [5.00–11.00]	8 [6–11]	-	7 [4–10]	0.009
Outcome					
Median time spent in ED (min) median [IQR]	144 [77–248]	160.5 [102.25–282.75]	69 [29.5–115.5]	155 [81–246]	0.001
Incidence of boarding *N* (%)	57 (13.9)	29 (19.6)	3 (3.8)	25 (13.7)	0.004
Mortality *N* (%)	137 (36.8)	62 (44.9)	21 (30.4)	54 (32.7)	0.043

**Fig. 5:**
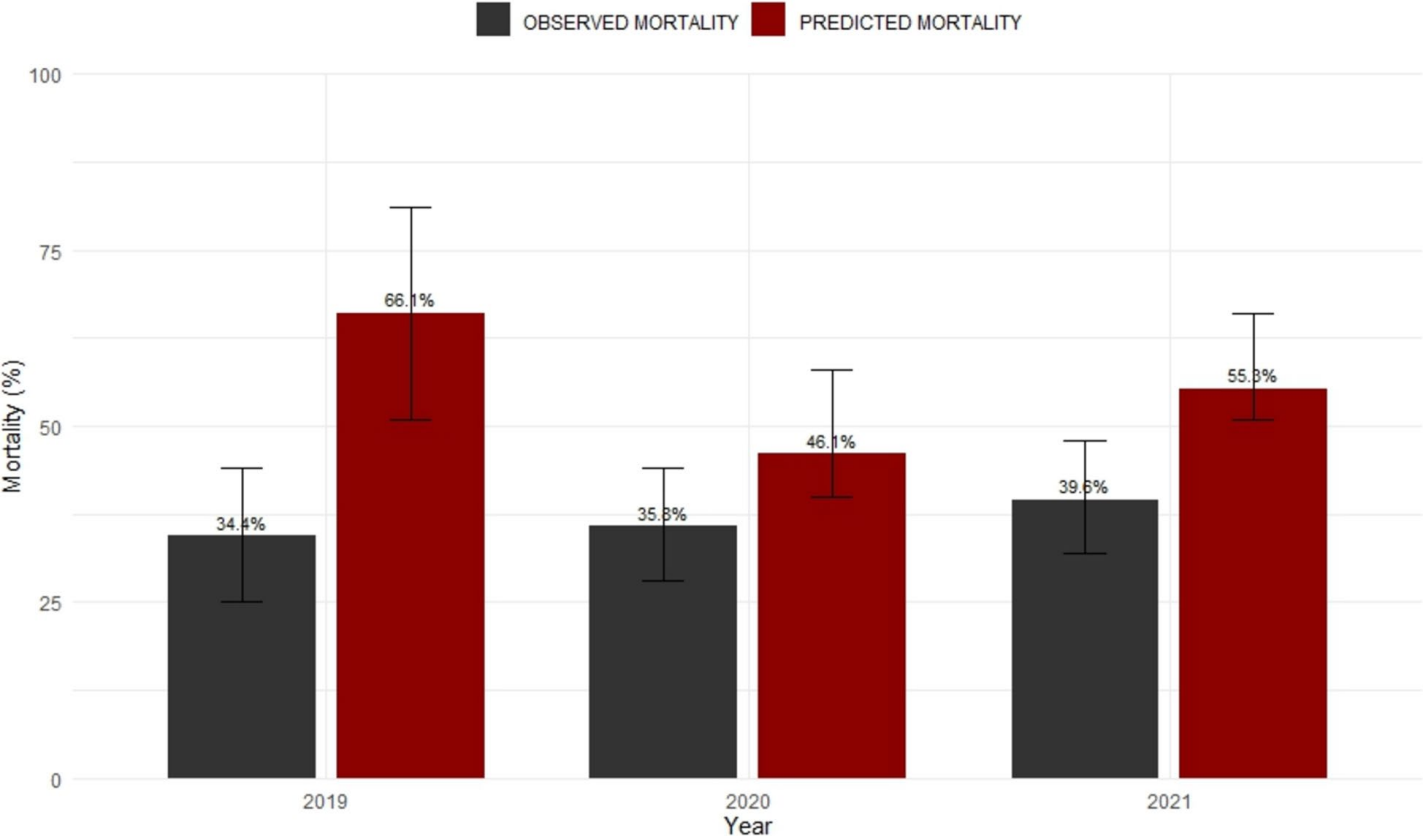
Comparison of predicted and observed mortality as a percentage in our sample and 95% CI error bars

The median SAPS II value was 54 points (*IQR* 40.5–69), with no difference during the study period (*p* = 0.15). Expected mortality based on SAPS II calculation was 55.3%, while the observed mortality reported for patients admitted to the CREM Unit was 36.8% (95% *CI* 31.9% to 42.1%, chi-square 21.53, *p* < 0.00001). “The observed mortality in 2019 was 34.4% (95% CI 25.5% to 44.5%), while the observed mortality in 2020 was 35.8% (95% CI 27.9% to 44.6%), and the mortality in 2021 was 39.5% (95% CI 31.3% to 48.4%).” The immediate mortality in the ED was 19.8%.The predicted mortality rate refers to the SAPS II calculation formula. The scores corresponding to the average SAPS II of the sample measured over the years were converted into estimated mortality rates (Fig. 5).

A predictive model referring to ED length of stay and mortality based on SAPS II is presented (Table 4). The sample consisted of 295 patients for whom both variables were available; however, 10 of these patients were transferred to other hospitals. Therefore, the multivariable model exploring the relationship between mortality and SAPS II was calculated on 285 patients, which is the cohort that was not transferred and for which outcome data were available. In terms of length of stay, admission to CREM Unit for worsening conditions was found to be associated with a lower time of stay and also when normalized for severity of conditions. In the predictive model, age and patient’s severity were associated with increased risk of mortality. On the other hand, variables such as admission to CREM unit for worsening conditions, gender, and triage priority were found to not be associated with mortality. Interestingly, among the independent variables, length of stay in the ED was not associated with an increased mortality (*p* = 0.399). Lastly, we performed a predictive model referring to ED length of stay based on SOFA (Supplementary Table 1). The predictive models were assessed using the variance inflation factor, which revealed that none of the predictor variables was correlated with each other. A sentence clarifying the assessment of multicollinearity has been added to the text.

**Table 4:** Predictive models. Emergency department length of stay predictive model with CREM Unit admission, demographic variables, SAPS II, and triage priority as dependent variables. Mortality predictive model with ED length of stay and SAPS II as dependent variables

ED length of stay			
		LOS (min)	
Predictors	Estimates	CI	p
Direct admission	Reference		
Worsening patient	−202.45	(−333.18, −71.7)	0.003
Worsening patient: SAPS II	3.32	(1.20, 5.44)	0.002
SAPS II	−0.47	(−2.14, 1.20)	0.579
Age	−1.38	(−2.91, 0.14)	0.074
Gender (F)	Reference		
Gender (M)	−33.19	(−84.23, 17.86)	0.202
Yellow code	Reference		
Red code	0.36	(−68.36, 69.07)	0.992
Higher green	−81.80	(−245.62, 82.01)	0.326
Lower green	36.24	(−69.61, 142.10)	0.501
*Observations*	295		
Mortality			
*Predictors*	*Odd ratios*	*CI*	*p*
(Intercept)	0.08	(0.02, 0.34)	0.01
Worsening patient	0.58	(0.31, 1.07)	0.082
Length of stay	0.97	(0.89, 1.04)	0.399
Gender (M)	0.78	(0.44, 1.37)	0.381
Age	1.02	(1.00, 1.04)	0.032
SAPS II	1.03	(1.02, 1.05)	< 0.001
Red code	0.62	(0.28, 1.34)	0.222
Higher green	2.39	(0.40, 19.89)	0.343
Lower green	0.47	(0.12, 1.66)	0.257
*Observations*	285		

## Discussion

Compared to international data, our institution registered a lower incidence of boarding (13.9%). Furthermore, over the study period, we saw an association between the decrease in boarding rate and an increasing trend in patients treated by the CREM Unit in the ED. This may be the result of progressive CREM integration in emergency department mechanisms and of personnel becoming more acquainted with inclusion criteria. A second relevant outcome of our investigation is that the CREM Unit promotes a timely and desirable treatment of acute critically ill patients in the ED, as evidenced by the observed 28-day mortality. The cohort of patients hospitalized after being managed by the CREM Unit had a 28-day survival rate of 63.2%, much higher than expected based on SAPS II.

Our predictive model, based on data from our institution, found mortality to be associated with severity indexes such as SOFA and SAPS II, but not with boarding time (Supplementary Table 1). This seems to be in contrast with international literature [13–15]. Nevertheless, it may be explained by a lack of standardization on the definition of boarding [3–6]. This limit precludes meaningful aggregation of data or comparisons between published results. An ED-Critical Care Medicine Boarding Task Force was set up, supported, in part, by the Society of Critical Care Medicine and the American College of Emergency Physicians. This task force recommends that ED-CCM boarding be defined as time spent in an ED (1) after the decision to admit to an ICU is made (existing ACEP boarding definition [16]) or (2) after 6 h in the ED (from ED arrival), whichever comes first. This is the definition we refer to in our work. This recommendation is based on recognition that boarding applies to patients for whom inpatient resources have been requested but are unavailable, but also that patient outcomes are worse for patients after 6 h of ED care even if no inpatient bed has been requested [3, 4]. On the other hand, while this is preliminary data and surely needs further investigation, the loss of association between boarding and mortality might reflect the CREM Unit’s effectiveness in providing critically ill patients with timely, appropriate, and intensive-level care. This is coherent with the concept that early, specialized intervention aimed at preventing further organ dysfunction and reducing risk factors that lead to patient death is of utmost importance during the first hours of patient treatment. Accordingly, Nguyen et al. also observed that Acute Physiology and Chronic Health Evaluation (APACHE) II and SAPS II calculated in the ED predicted mortality and concluded that care provided during the ED stay for critically ill patients significantly impacts the progression of organ failure and mortality [17].

Taking a deep insight into ED length of stay of our patients, it has to be underlined that it is mostly owed to the many procedures directly provided in the emergency department, ranging from simple bronchoscopy or dialysis to extracorporeal cardiopulmonary resuscitation (ECPR) on refractory cardiac arrests. The provision of such treatments to nonsurgical patients might explain the very low impact that boarding has on mortality. On the other hand, there are patients who require surgery or emergency interventional radiology procedures that have inherently limited boarding, thanks to a higher availability of the appropriate destinations. In some cases, such as intoxicated patients with brief need of ventilatory support, or simple airway procedures such as tracheal cannula repositioning, the prompt intervention of the CREM Unit made a therapeutic de-escalation possible. Thus, the treatment provided in the ED eliminates the need for an intensive care bed and the associated possibility of boarding as an intensive care patient.

Concerning the boarding time to ICU, Chalfin et al. using a stepwise logistic model found delayed admission, advancing age, higher APACHE II score, male gender, and diagnostic categories of trauma, intracerebral hemorrhage, and neurologic disease to be associated with lower hospital survival. The authors emphasize the importance of detecting the most fragile patients to whom a delayed admittance to the ICU would be particularly harmful. Literature seems to agree that a delayed admission to the ICU impacts outcome in terms of length of hospital stay, worsening patient conditions as well as morbidity and mortality. This can be avoided with a different organization in critically ill patients’ management. In our study, we registered an ICU boarding of 20.2%. This is higher than that registered for surgical patients directed to OR, but no association with mortality has been detected using subgroup analysis. This further supports the role of intensive-level treatment provision in the ED in improving patients’ survival, and our findings are consistent with Gunnerson K. J. et al. [18].

To our knowledge, this is the first study reporting data on patients’ outcomes treated by a CREM Unit within an “ICU without walls” model and has been conducted on a large sample and wide range of cases. However, some limits are still present. First, the single-center nature of observations limits the external validity of our findings. San Giovanni Bosco Hospital is configured as a second-level community hospital; it is equipped with all surgical specialties, and thus, it acts as a referral hospital (hub) for the northern area of the province of Turin. It has a renowned appeal for acute medicine, and our low-boarding low-mortality emergency department data may be due to a more complex synergy of factors. Second, it is difficult to weigh the effect of COVID-19 occurrence during the 2020 and 2021 observations, as our area was deeply affected by the pandemic. Third, we did not compare the output of the CREM Unit established in 2019 to a historical cohort, since a less formalized activity of intensivists in the ED has always been present, with a progressive shift towards the described model. Therefore, the debate regarding the most functional model of intensive care delivery remains open, especially regarding the specialties involved and their competencies [19–23]. A recent study by the European Society of Anaesthesiology and Intensive Care (ESAIC) highlights the growing integration of perioperative medicine, ICM, and CREM in Europe, emphasizing a multidisciplinary approach for patient safety and improved outcomes [24]. In this regard, the authors therefore highlight the need to clarify the roles and responsibilities of the different specialties engaged in emergency care. Interestingly, there is a lack of literature about cooperation between these figures. Treatment of critically ill patients in the ED should involve a variety of medical or subspecialties, such as intensivists, EPs, and anesthesiologists. To address these unmet acute critical care needs, several institutions in the USA have established resuscitative care units (RCUs). While each unit has been designed to meet its specific institutional needs, all RCUs focus on providing timely and specialized care to critically ill patients with diverse conditions and pathophysiology, engaging different medical specialties encouraging cooperation [10]. Mitigation strategies were organized into three focus areas: (1) ED solutions, (2) hospital solutions, and (3) ED-based resuscitative care units (RCUs) [4]. Different mitigation strategies have been adopted; a “resource intensivist” model was proposed in 2012 and is based on the constant presence of a physician trained in intensive care in the ED. Two ED-ICU models have been described: the “hybrid” and the “stand-alone ED-ICU.” The hybrid model enables management of critically ill patients beyond the first hour after resuscitation, constituting a hybrid “resuscitation bay and ED-ICU” (RED-ICU) [2]. This model was utilized in a study including critically ill patients who required time-sensitive surgical interventions. The authors reported that implementation of the model facilitated transfer to the operating theater or ICU and improved patient outcomes.The stand-alone ED-ICU model consists of an isolated unit within the ED containing ICU beds and appropriate equipment. A stand-alone ED-ICU, termed the emergency critical care center (EC3), has been created and established at the University of Michigan University Hospital. The University of Michigan EC3 was the site of a large observational retrospective cohort study from 2012 to 2017. The authors concluded that ED-ICU implementation was associated with lower 30-day mortality and reduced rates of ED to ICU transfer. This model might not be cost-effective due to the need for de novo creation of ICU beds. However, randomized controlled trials along with cost-effective analyses are needed to conclude whether ED-ICUs have a significant impact on patient care [2]. These mitigation strategies are starting to gain more importance and should be considered in certain hospital scenarios, but further investigations are needed to better evaluate the impact of roles, rather than competencies or organizational models on patients’ outcomes.

We believe this model is easily replicable, especially in the European hospital context, because it does not involve changes in hospital structure nor demands more hospital personnel or a drastic change in hospital protocols. The model we suggest is based on a shift in paradigm towards an “ICU without walls” concept and concerns and optimization of hospital resources in terms of roles and responsibilities avoiding overloading emergency physicians and in terms of procedures directly provided in the emergency department preventing boarding and burden over certain specialties. The longitudinal approach guarantees for the pathway of critical emergency patients a direct and seamless translation from specialty expertise into the ICU. Especially after the competencies of European anesthesiologists have been redefined, our model should be easily incorporated in any European hospital context. CREM Unit focuses on providing timely and specialized care to critically ill patients with different conditions and pathologies, relying on the use of a multidisciplinary and multi-professional approach for such complex patients collaborating,if needed, as needed with ward and ED nurses and physicians.

This study has some limitations. Firstly, it is an observational study, and further research is needed to better define the impact of boarding on the mortality of patients managed by the CREM Unit, which is based on the presence of an intensivist in the emergency department. Moreover, it was not possible to collect certain data related to 28-day mortality and SAPS II scores. In fact, the SAPS II score is missing for patients who were transferred from hospitals outside the local healthcare network; this implies that the SAPS II score, which would normally be calculated upon the patient’s arrival at the first emergency department, could not be determined due to a lack of data. As a result, the study may be affected by a selection bias related to the different characteristics of patients transferred from other hospitals (e.g., more severe conditions, different socio-economic backgrounds, different treatments, and differing timelines). In conclusion, the potential impact of the COVID-19 pandemic on the study remains unknown.

## Conclusion

Contrary to what has previously been reported in the literature, our study showed that the CREM Unit boarding time is not associated with an increase in mortality under certain conditions. This finding highlights the effectiveness of the “ICU without walls” model and of our CREM Unit in rapidly treating critically ill unstable patients. We believe the strength of the proposed model lies in being an “ICU-based” model, capable of guaranteeing an early resuscitative approach, individualization of invasive monitoring, and early medical-surgical therapeutic strategies from the perspective of a longitudinal approach.

## Supplementary Information

Supplementary Table 1: Emergency department length of stay predictive model with CREM Unit admission, demographic variables, SOFA, triage priority as dependent variables.

## Data Availability

No datasets were generated or analysed during the current study.
