# Supplementary material for: Critical emergency medicine unit: a new model to mitigate critically ill patient boarding in emergency department
Source: J Anesth Analg Crit Care. 2025 Jul 10;5:42. doi: 10.1186/s44158-025-00262-x (PMC12247336; doi:10.1186/s44158-025-00262-x)
Supplement: Supplementary file 1 — Supplementary Table 1: Emergency department length of stay predictive model with CREM Unit admission, demographic variables, SOFA, triage priority as dependent variables. [file 44158_2025_262_MOESM1_ESM.docx]

| **ED Length of stay** |  |  |  |  |
| --- | --- | --- | --- | --- |
|  |  |  | *LOS (min)* |  |
| *Predictors* |  | *Estimates* | *CI* | *p* |
| Direct admission |  | Reference |  |  |
| Worsening patient |  | -146.21 | (-259.56; -32.86) | **0.012** |
| Worsening patient:SOFA |  | 16.10 | (3.59; 28.61) | **0.012** |
| SOFA |  | -2.49 | (-12.04; 7.05) | 0.607 |
| Age |  | -0.94 | (-2.40; 0.52) | 0.207 |
| Gender (F) |  | Reference |  |  |
| Gender (M) |  | -30.75 | (-82.17; 20.68) | 0.240 |
| Yellow code |  | Reference |  |  |
| Red code |  | 6.66 | (-62.36; 75.67) | 0.850 |
| Higher green |  | -57.63 | (-221.5; 106.31) | 0.490 |
| Lower green |  | 41.48 | (-65.20; 148.17) | 0.445 |
| *Observations* |  | 295 |  |  |
